# Application of Fawcett’s Criteria in Theory Evaluation

**DOI:** 10.1177/08943184221131966

**Published:** 2022-12-26

**Authors:** Steve Iduye

**Affiliations:** 1 Assistant Professor, Cape Breton University, Nova Scotia, Canada

**Keywords:** Donabedian framework, health outcomes, nursing informatics, theory evaluation

## Abstract

Nursing informatics is an emergent field of practice, as are the conceptual and theoretical frameworks that underpin research in this field of practice. In research, theoretical frameworks serve as structured roadmaps that connect various concepts and propositions in a field of study. Therefore, building theoretical frameworks in nursing informatics requires evaluating relevant knowledge from other disciplines that intersect with nursing informatics to justify its relevance and applicability. Fawcett’s criteria provide feasible approaches for evaluating middle-range theory. Consequently, the prominent health program framework popularly referred to as the Donabedian Healthcare Quality Framework is significant to nursing informatics research and projects.

The field of health informatics intersects with cognitive, information and computer science. Nursing informatics integrates clinical information and knowledge of technology to improve people’s and communities’ health while reducing costs (Canadian Nurses Association, 2020). Since nursing research and practice support the provision of quality care and continuous improvement of health services, the use of the Donabedian Healthcare Quality Framework (DHQF) provides a systematic and practical approach for evaluating health services and the quality of care the patients receive (Donabedian, 1966). The development of a discipline-specific theory in nursing informatics is invaluable in building the science and theoretical roadmaps upon which informatics research will thrive. Effken (2003) postulated that theory drives the progress of science in any discipline. In other words, without a discipline-specific theory as a guide, research will focus on specific problems rather than the underlying causes of the issues (Verran, 1997). Given the shortage of theory specific to nursing informatics, this new field’s growth relies on borrowed theories or atheoretical stances. For example, in the last two decades, a review of published literature by Peterson and Bredow (2017) from January 1994 to June 1997 revealed 22 middle-range nursing theories.

In addition, Im (2018) found a literature search for middle-range nursing theories from 2008 to 2018 on PubMed, PsycINFO, and CINAHL revealed 23 middle-range theories, none of which were specific to nursing informatics. These literature searches reveal a dearth of theoretical frameworks for application to nursing informatics research. In support of building theoretical momentum in the nursing informatics field of practice, it is imperative to explore applicable theories from various disciplines, such as cognitive, information, and computer science, that intersect with nursing informatics. The future of nursing informatics research seems dependent on relevant borrowed theories until the field gains its theoretical momentum. A suitable alternative is to test the applicable borrowed theories or modify their applications to the nursing informatics research.

## The Argument for Modification of Theory

There are two schools of thought regarding the use of borrowed theory in nursing. Some scholars were content to draw nursing theories from existing sciences (Risjord, 2009a) because they believe that other practice fields have developed the sound knowledge needed in nursing (Gunter, 1962). The other school of thought thinks any borrowed theory should be tested and modified before it applies to nursing issues or practice (Ellis, 1968; Risjord, 2009a). Otherwise, the borrowed theory may not apply to nursing and its particular problems and needs (Bluhm, 2014). Although this debate dates back to the 1960s, it still poses serious concerns for many nurse scholars today (Bluhm, 2014). At this state of theory development in nursing informatics, the latter serves the new field of practice better. Modification of relevant borrowed theories requires understanding the differences in meaning between the borrowed theory and the nursing field in terms of the concepts and propositions. Otherwise, the borrowed theory is liable to create ambiguities in the nursing lexicon. A particular discipline is likely to have unique definitions of concepts that would have a different meaning when applied to another profession. Such differences are one of the dangers associated with borrowed theories in nursing. Bluhm (2014) and Risjord (2009b) argued that nurse researchers and theorists should not incorporate borrowed theory into nursing without alteration or modification. This modification of borrowed theories in nursing, however, must be done with caution. First, the nurse theorist or researcher needs to identify the concepts in the borrowed theory. Second, a comprehensive concept analysis in those adopted theories must be conducted; either through theoretical or colloquial approaches (Risjord, 2009b). Third, a thorough evaluation of the entire borrowed theory against a set of theory criteria is required to ensure applicability to nursing problems (Fawcett, 2005). Once these requirements are successfully met, the borrowed theory can be tested in a clinical setting to validate its use in real clinical nursing situations. Risjord (2009b) and Fawcett (2005) rightly suggested that highlighting the differences in the meaning of concepts of theory allows comparison with the evaluation criteria to establish relevance and validity. Applying concepts to nursing research without this initial analysis can result in ambiguities that make studies incommensurable and impractical (Risjord, 2009b).

## Applying Fawcett’s Criteria to Quality Healthcare Framework

Avedis Donabedian proposed the Healthcare Quality Framework (HQF) in 1966 to evaluate medical care and healthcare systems’ quality. For over five decades, the HQF has been the gold standard for healthcare quality outcome assessment and researchers who appreciate this groundbreaking research work continue to apply it in health outcomes research (Salzer et al., 1996). Although Donabedian was a medical practitioner by training and practice, his biomedical orientation to patient care formed the primary principle of the framework. Donabedian considers the health system as continually evolving and needing consistent evaluation by examining three categories: structure, process, and outcomes (Donabedian, 1966).

The term “Structure” encompasses the material resources of a care setting. These include the equipment and tools, budget and financial capacity, facilities and human resources (both the number and qualification of personnel), methods of hospital reimbursement, and peer review (Donabedian, 1988). “Process” refers to a series of activities and actions within the health systems that guide the decisions made in diagnosis, treatment, and recommendations for patients (Donabedian, 1988). “Outcome” refers to patients’ health status and their level of satisfaction after receiving care (Donabedian, 1988). According to Donabedian (1966), the following three principles explain the operationalization of the framework in evaluating healthcare systems: first, the researcher should understand the relationships of causality between structure and process and between process and outcome, concerning the proposed research or health program, and second, the framework builds on the assumption that good structure and process increase the likelihood of a good outcome. Third, Patient satisfaction, an essential component of outcome measurement, is inextricably linked with the clinical competencies of care providers.

## Conceptual Evaluation

Fawcett’s (2005) criteria for theory evaluation imply an objective and nonjudgmental description of the theory and addresses how the theory meets specific evaluation criteria. The criteria for evaluating theory are significance, internal consistency, parsimony, testability, empirical adequacy, and pragmatic adequacy. According to Fawcett (2005), significance explains how the framework explicitly addresses the metaparadigm concepts, philosophical claims, and conceptual model. The HQF is not posited as a philosophical framework but a practical framework for evaluating the quality of care outcomes. Nevertheless, the concepts of HQF (structure, process and outcomes) are consistent with the person, health, and environment in the nursing metaparadigm. Nurses are obligated to provide quality care within the frame of ethical considerations in healthcare. Korhonen et al. (2015) study discussed the significance of HQF and nursing metaparadigm in technology and healthcare ethics. The authors used a caring science and integrative approach to examine technology’s concept and the meaning of ethics within the nursing literature. The literature search strategy included Medline and CINAHL databases for articles written in English and published in 2000-2013. The authors categorized technology as a process, a service, and a device or product. Ethics was less evident in caring, and nursing literature described ethics as evaluating risks, benefits, or unsolved problems. There was difficulty connecting the use of health technology, ethical considerations and fundamental aspects of caring science. Consequently, the authors concluded that current models that promote safe and ethical care are insufficient, and research into methods and processes and additional legislation must be undertaken to ethically integrate technology and caring science (Korhonen, Eriksson, & Nordman, 2015). Because nursing informatics has not provided an over-arching model or framework for the ethical use of technology in patient care, it is imperative to exercise caution when using HQF in digital related research.

Internal consistency criterion requires all constructs of the theory to be congruent, including the philosophical claims, conceptual model, concepts, and proposition (Fawcett, 2005). The concepts of the HQF are linear but interactive. The concepts do not stand alone but are in constant interaction with each other; for example, the interaction between structure and process determines the outcome. This interaction between structure and process of care corresponds to Fawcett’s (2005) criterion of parsimony, which, she explains, clarifies the proposition and concepts of a theory without oversimplifying the phenomenon of interest. For example, in Lindgren and Andersson (2011) prospective study of 95 patients and 120 personnel in the medical and surgical wards at a hospital in Sweden, the researchers examined the concepts of Donabedian’s Structure–Process–Outcome triad (S–P–O triad) to develop a measuring instrument for care quality. Testing the concepts of the instrument for construct validity and internal consistency, the authors performed factor analyses in two steps and Cronbach’s alpha coefficient, respectively. The results showed a logical distribution of concepts or variables and internal consistency that was good in both factor analyses. These results indicate that Donabedian’s Structure-Process-Outcome triad is suitable in clinical practice for measuring the quality of nursing care (Lindgren and Andersson, 2011).

For the testability criterion, the middle-range theory concepts must be observable with appropriate instruments to give credibility to the evaluated theory or framework (Fawcett, 2005). For example, the HQF is narrower in scope, with a less abstract focus (i.e., structure, process, and outcomes). The framework also comprises concepts and propositions for more specific phenomena, with the view that nursing realities are appropriate for empirical testing (Peterson & Bredow, 2017). In Gardner et al. (2014) study that evaluates nurse practitioner service’s safety and quality using the audit framework of the structure, process, and outcome. The authors collected a range of data through stakeholder surveys (N=36), in-depth interviews (11 patients, 13 nurse practitioners) and health records data on service processes. Their result indicates that adequate and detailed preparation of Structure and Process is essential for successful service innovation implementation. In addition, multidisciplinary team also accepted the addition of nursing practitioner service and nurse practitioner clinical care was effective, satisfactory and safe from the perspective of the clinician stakeholders and patients. This study demonstrates that the concepts of HQF could be observed and measured with a mixed-method design.

Empirical adequacy is used to ascertain the congruence between theoretical assertions and empirical evidence. All constructs of the HQF, as discussed previously, intersect with the nursing metaparadigm at the level of patient care, the processes involved in providing or receiving healthcare, and the outcome of care. According to Fawcett (2005), the similarity between the constructs of the theory and empirical evidence indicates empirical adequacy. Depending on the researcher’s chosen methodology or paradigmatic disposition, the HQF can be applied to qualitative or quantitative data to understand the relationships between the constructs of the theory.

Finally, pragmatic adequacy refers to how the theory applies to nursing, the feasibility of implementing nursing practice derived from the theory, and the measurement of outcomes in terms of the theory’s problem-solving effectiveness (Fawcett, 2005). At the intersection of nursing and informatics, the HQF provides a platform for understanding how health technology and data-driven clinical decisions will benefit patients. For example, Piscotty, Martindell, and Karim (2016) conducted a descriptive study using secondary data from 140 student participants in RN or BSN programs to evaluate their use of social media in the workplace. The authors applied the HQF as a guide to their study as follows: if nurses used social media at work (structure) to gather and implement clinical data (process), the result could be beneficial to the patients (outcome). The authors concluded that social media use in the workplace was prevalent among nurses and positively and negatively affected patient care. The authors concluded that social media use in the workplace was prevalent among nurses and positively and negatively affected patient care. The authors also found that the HQF was invaluable to the conduct of nursing research for measuring the effectiveness of nursing services in a patient population.

## Potential Gaps in the Donabedian Healthcare Quality Framework

Healthcare systems are complicated by many interdependencies between the structural design, and the implementation of work processes. For example, the nursing home facility can not function in isolation of nurses, residents and different processes involved in delivering care. Several processes depend on each other for residents to receive quality care. These multiple interdependencies inevitably influence patients’ outcomes (Shah, 2019). Therefore, to achieve continuous improvement in patient outcomes, it is essential to conduct a thorough examination of the relationships between the structure and the processes of care. Although there are many advantages to using HQF in nursing informatics research, the framework also has gaps that pose potentially severe drawbacks for researchers. First, HQF does not establish how to determine what constitutes the quality of structure and process. What constitutes the quality of structure and process is multi-faceted. Salzer et al. (1996) recommended the use, by many health organizations, of quality measurements based on standard operating procedures, report cards, and practice guidelines developed through literature reviews, consensus, and expert panels. However, Battles (2006) refuted Salzer et al. ’s stance on the extent to which these measurements translate into quality outcomes for the residents. Battles argued that the healthcare delivery system is provider-centric; in other words, the system focuses on the convenience of the care provider rather than putting the residents at the center of clinical decision making and care delivery. Cho (2001) acknowledges Battles’ assertion, proposing that managerialism as a corporate style in healthcare systems influences the organizational structure and leads to operational failures.

Another critical gap in HQF is the confounding factors. The determination of quality outcomes for residents might not depend solely on the relationship between structure and process but may be influenced by the presence of confounding variables. This gap is a direct violation of the criterion for internal consistency. Inarguably, there is an assumption that a relationship exists between HQF structure, process, and outcome. In other words, the reliability of the importance of HQF structure and process remains questionable, because the extent to which these quality indicators link to outcomes is unknown (Salzer et al., 1996). Confounding variables are not limited only to the conduct of research. For instance, in a long-term care setting, a recent scoping review found that a clinical tool called the international resident assessment instrument (interRAI LTCF) provides a viable way of collecting clinical data for assessment, identifying at-risk residents, supporting the formation of an appropriate care plan, and improving the quality of care at the facility level (Iduye et al., 2021). However, other factors like how well the staff uses the assessment tool, adequate nutrition of residents, residents’ comorbidity, and interdisciplinary support in long-term care homes might not be appropriately accounted for as contributors to the residents’ positive or negative health outcomes. Without controlling factors such as these that contribute to health outcomes, the care plan generated for the resident is not enough to ensure quality outcomes for the resident.

Furthermore, controlling the confounding variables is a reliable and valid way to increase the internal consistency and promote congruence between theoretical assertions and empirical evidence of DHQF as middle-range theory. Confounding factors can mask an actual association between the process of treatment and outcome or falsely demonstrate an association where none exists (Skelly, Dettori, & Brodt, 2012). These confounding variables need to be reviewed continuously and brought forward in the care planning to facilitate the understanding of the interaction between specific components of structure and process that elicit the desired outcome. Knowing what constitutes confounding variables in nursing care planning for residents informs multiple approaches to effective and efficient care. As long-term care residents with chronic diseases require more nursing care, understanding the nature of confounding variables in the provision and delivery of care increases nurses’ professional ability to meet residents’ holistic health needs.

Lastly, outcome quality measurement should be a continuous process for long-term care residents. Outcome quality measurement is a reputable characteristic of HQF that promotes the internal consistency and significance criteria of HQF as a middle-range theory. Knowing that long-term care facilities operate under different management styles that may influence the provision of care, and residents’ health outcomes, the need for continuous measurement of care quality and residents’ health outcomes are invaluable. In most LTC, the provision of quality care to the residents has suffered from insufficient government funding and neglect, all of which pervade long-term care and its administration. Moreover, there is high turnover among registered nurses (RNs), and retention is difficult; several attempts to address the situation have not produced positive results (Collier & Harrington, 2008). Nurses have an obligation never to cease to meet the clinical needs of resident populations. Chronic disease management in residents of long-term care facilities require the iterative and continuous interaction of different nursing care for a sustainable quality outcome throughout the resident’s life in the facility. Due to the residents’ clinical needs that require ongoing nursing care, it is safer to monitor a specific health outcome at a time—knowing that the residents might not have the capacity to participate in multiple care processes simultaneously. This approach may seem lengthy and time-consuming for nursing staff. However, it aggregates a consistent and specific outcome to determine whether the changes lead to a decline or improvement in one particular overall outcome (Shah, 2019).

## Building New Propositions in Healthcare Quality Framework

The term quality care is often interpreted by residents to mean satisfaction with nurses’ care, as indicated in HQF. At the same time, it is tempting for nurses to consider that changes in residents’ health status after the care provided are an accurate indicator of care quality outcomes. The new proposition that will strengthen HQF use in nursing informatics research is resident centred care approach. Resident-centred care is care organized around the resident, where providers regularly work with resident and their decision-makers to identify and satisfy a full range of resident needs and preferences (Picker Institute, n.d.). As highlighted in the Picker Institute document, the principles of patient or resident-centred care are divided into the following categories: (a) respect for patients’ values, wishes in terms of the involvement of family and friends, preferences, and expressed needs; (b) coordination and integration of care by the nurses throughout the illness; (c) information, communication, and education about the details of the disease—provided to the residents as well as to families and those who act on a resident’s behalf; (d) psychological and emotional support to alleviates fear and anxiety; and (e) prompt initial access to care and transition or continuity of care. In contrast to the linear pattern and relationship of the HQF concepts, patient or resident-centred care is a multidimensional approach to solving residents’ clinical needs. It empowers patients, residents and their families to make clinical decisions in the best interests of residents. With the patient-centred care approach, the Structure, Process and Outcomes of care are modified to accommodate the patients and residents as partners in decision making, care planning and implementation. By incorporating the concept of patient or resident-centred care into the Structure, Process, and Outcomes, patients and residents will make contributions to their care, with proper documentation for all clinical staff to follow.

## Contextualizing the Propositions

Residents or their families’ satisfaction should be a predictor of what constitutes the quality outcome of care in long-term care settings. As recipients of healthcare, patients and residents provide indispensable feedback that could be used to help improve the overall quality of the management of healthcare systems. The satisfaction component of quality care outcomes addresses the interpersonal relationship that is part of the care process and informs changes in the resident’s health status (Donabedian, 1988). Changes in resident health status are, in fact, relative to his or her definition of quality outcomes. For example, suppose a nursing home resident’s care plan specifies, in the advance directives, a preference for no cardio-pulmonary resuscitation (CPR) and peaceful death after a protracted illness. To this resident and her family, the quality care outcome could be expressed as a peaceful death during palliative care if there was no CPR given. Similarly, for residents who could tolerate living in a long-term care facility because the nurses support them to feel at home, this would influence both the residents and the family’s satisfaction. These examples show the need to include resident satisfaction as a predictor of quality care improvement. How to promote residents’ satisfaction is attributed to the level of engagement in care processes. Evidence suggests that patients/residents who are well involved in their health care processes experience better health outcomes and incur lower costs (James, 2013).

To make residents’ engagement relatable to both the residents and care providers, Carman et al. (2013) developed a continuum of engagement in direct care that involves consultation, involvement, partnership and shared leadership. In consultation, the residents receive information about a diagnosis or treatment from care providers. Involvement is to prioritize residents’ needs by asking about their needs and preferences in the treatment plan. Shared leadership involves treatment decisions based on residents’ preferences, medical evidence, and clinical judgment of care providers. Further, Carman et al. (2013) expanded the continuum of engagement to include research and policy making for the resident population. During the consultation, the public agency will conduct focus group interviews with patient/resident population to ask opinions about a healthcare issue. Involvement includes using the residents or patients’ recommendations about research priorities to make funding decisions by the public agency. Shared leadership promotes a platform where patients have equal representation on the agency committee that makes decisions about allocating resources to health programs.

## Conclusion

In this discussion paper, an evaluation of HQF and its applicability to nursing informatics has established the framework as a middle-range theory. The framework’s reliability will always constitute challenges to the knowledge users if the potential gaps in the framework are not identified and defined. This paper draws on the nursing literature to justify framework usability and develop propositions based on resident-centred care to improve patients’ quality outcomes in nursing informatics research. The Health Quality Framework provides a feasible and realistic approach to healthcare program planning and evaluation. The evidence presented in this paper on the scarcity of nursing informatics middle-range theories and conceptual frameworks opens a crucial discussion on the need for borrowed theories in nursing informatics. The danger that lies ahead in nursing informatics is the possibility of an atheoretical stance or indiscriminate use of untested borrowed theories as alternatives.

An adopted framework like HQF provides a practical way of evaluating healthcare quality outcomes in research and practice settings. However, this framework needs to contextualize the core metaparadigm of nursing adequately. Otherwise, the HQF would only project its original meaning onto nurses. The HQF presents some issues of concern, as it does not establish how to design a quality structure and process in healthcare. The lack of definition of what constitutes quality design of healthcare complicates the nurse researcher’s entire process of evaluation. Without an idea of what quality means in healthcare design, the measurement of HQF processes of treatment and outcome quality could be compromised. Another problem that HQF presents is a lack of objectivity regarding how confounding factors or variables could influence healthcare outcome quality. This lack of objectivity undermines the internal validity of HQF.

The design of HQF for nursing informatics research should reflect resident-centred care, which is the care organized around the patient, allowing care providers to work with the residents, considering their preferences. Moreover, the ambiguities between HQF structure, process, and outcomes are successfully addressed, with resident-centred care driving various constructs of HQF. The use of HQF in nursing, therefore, should be informed by patient or resident-centred care components to establish the design of HQF structure and process quality in healthcare. It is vital for nursing informatics researchers to know that; their research findings should add significant values to the patient population with patient-centred care that would connect the structure of care, the process of treatment, and the quality health outcomes for the residents.
